# Clandestine abortion causing uterine perforation and bowel infarction in a rural area: a case report and brief review

**DOI:** 10.1186/s13104-016-1926-5

**Published:** 2016-02-16

**Authors:** Carlson B. Sama, Leopold Ndemnge Aminde, Fru F. Angwafo

**Affiliations:** Islamic Medicalized Health Centre, Babessi, Cameroon; Clinical Research Education, Networking and Consultancy (CRENC), P.O. Box 3480, Douala, Cameroon; School of Public Health, Faculty of Medicine and Biomedical Sciences, University of Queensland, Brisbane, Australia; Gynaeco-Obstetric and Paediatric Hospital, Yaoundé, Cameroon

**Keywords:** Unsafe abortion, Uterine perforation, Bowel injury, Rural, Cameroon

## Abstract

**Background:**

An unsafe abortion is defined as a procedure for terminating an unintended pregnancy carried out either by a person lacking the necessary skills or in an environment that does not conform to minimal medical standards or both. Majority of these unsafe abortions are carried out in rural areas of developing countries, usually by unskilled persons who do not have proper knowledge of the anatomy of reproductive organs and in unhygienic environments thus leading to various complications.

**Case presentation:**

We discuss the case of a 21 year old female who presented in septic shock after she underwent an unsafe abortion of an 11 weeks pregnancy with uterine wall perforation and bowel injury that required resection.

**Conclusion:**

Unsafe abortion is an important public health problem which accounts for a significant cause of maternal mortality and morbidity in resource poor countries. A high index of suspicion of clandestine abortion with ensuing complications should prevail when faced with a woman of child bearing age with the triad of vaginal bleeding, amenorrhea and pelvic sepsis.

## Background

Unsafe abortion is a public health challenge as approximately 13 % of pregnancy-related mortality worldwide is due to clandestine abortion. It remains the principal cause of a range of short- and long-term health complications in women [1, 2]. Worldwide, estimates suggest that over 19 million unsafe abortions occur yearly, with about 10–50 % seeking medical care for complications and about 47,000 women dying due to unsafe abortions [2–5]. There is a disproportionate global distribution of unsafe abortions with Africa being the most affected continent. This in part might be explained by the fact that most African countries have restrictive abortion laws, limited access to reproductive health services and high unmet needs for family planning services [6]. With the exception of cases of rape or incest, abortion is still illegal in Cameroon which explains why most unwanted pregnancies end up in clandestine abortions [7, 8].

In rural Cameroon, induced abortions are usually performed by unskilled personnel including inexperienced birth attendants and nurses in unhealthy environments with ensuing complications which may involve injury to the gastrointestinal tract.

## Case presentation

**Fig. 1:**
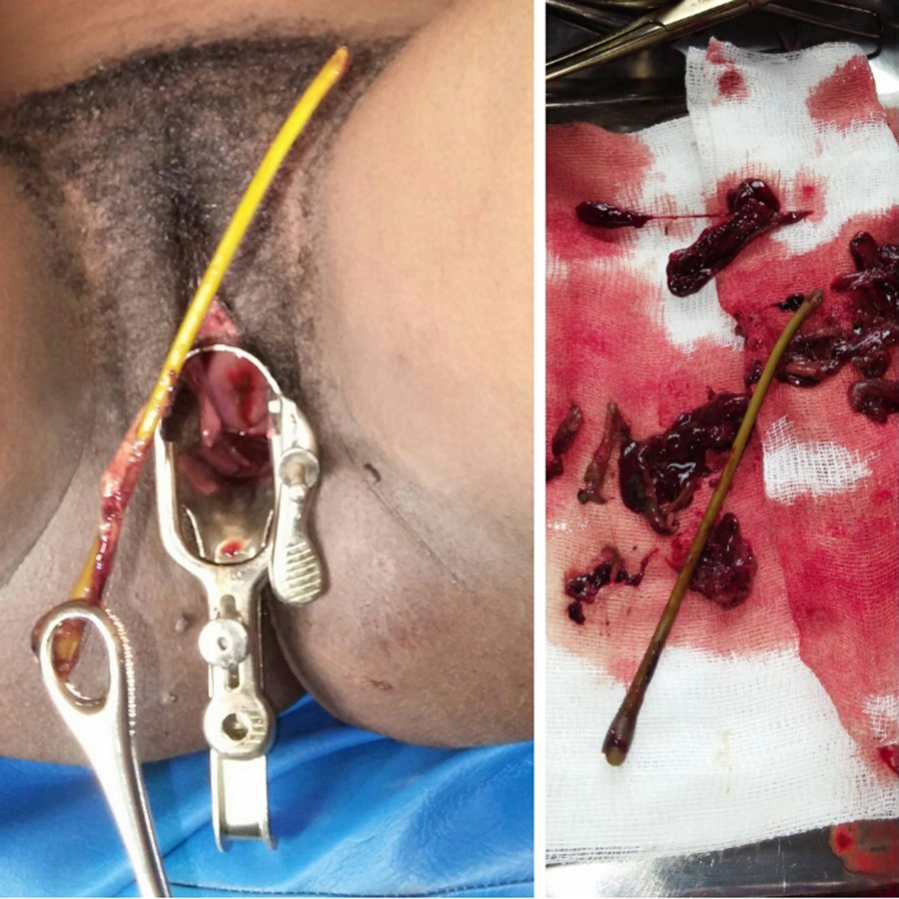
Cassava stalk and necrotic debris immediately after removal

A 21 year-old female who presented at 11 weeks amenorrhea with complaints of lower abdominal pains and persistent vaginal bleeding mixed with dark clots for the last 4 days. She is gravida 0. Despite careful inquiry, she denied being pregnant or any attempts to temper with a possible pregnancy. On examination, she was dehydrated, had a temperature of 38.7 °C, respiratory rate of 26 breaths per minute, pulse rate of 115 beats per minute and her blood pressure (BP) was 90/60 mmHg. There was mild superficial tenderness on palpation of the suprapubic region. The uterus was bulky and palpable 5 cm above the symphysis pubis. Bowel sounds were present but hypoactive. On vaginal examination, the introitus was blood stained and the congested cervix was 4 cm dilated. There was cervical motion tenderness and no active bleeding was noted. A solid thin stick-like material was felt protruding via the open cervical os. It was easy to extract as it slip pass the os and fell into the vaginal cavity. We carefully picked it up and identified the 14 cm long stick as a cassava (*Manihot esculenta*) stalk (Fig. 1). The examining gloved finger was stained with abundant foul smelling necrotic tissue. Only then did the patient admit to knowing she was pregnant several weeks back and she emphasized it was a deliberate attempt to terminate the unplanned and undesired pregnancy. The stalk was inserted 3 h prior to the onset of symptoms by a resident of the remote village, who is renowned as a local traditional abortionist. A urine specimen tested positive for pregnancy and haemoglobin level was at 8 g/dl. A total white cell count was 13,500 × 10^3^/μl with absolute neutrophil count of 87 %. No imaging modality was done due to financial constraints. A working diagnosis of septic shock following an induced abortion was made. Intravenous fluid resuscitation and empiric parenteral antibiotics were initiated.

**Fig. 2:**
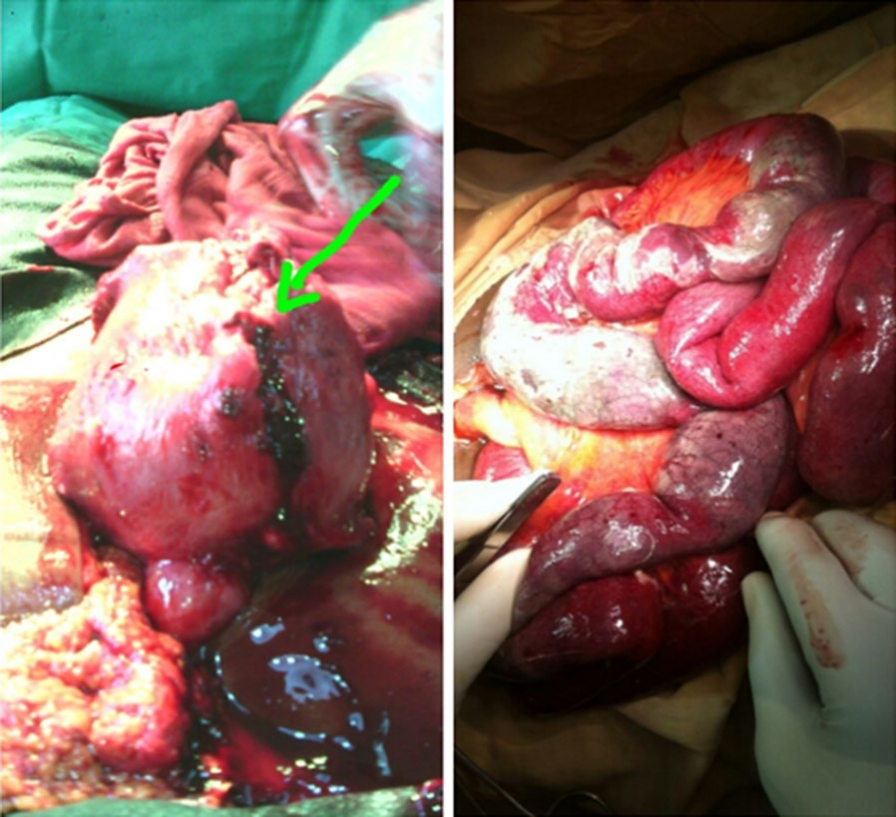
Haemoperitoneum, area of uterine fundal perforation and necrotic bowel

Four hours post admission, there was frank tenderness, rigidity and rebound tenderness on palpation of the suprapubic region. Bowel sounds were absent. Based on clinical evaluation, an emergency laparotomy was done via a midline infraumbilical vertical incision. Intra operatively, the peritoneal cavity contained about 1.5 l of blood mixed with feaculent material. We found a segment of necrotic small bowel which was adherent to a 2 cm wide perforation at the fundus of a bulky and edematous uterus (Fig. 2). A 3 cm long piece of the cassava stalk was found linking the perforated uterus to the necrotic bowel. This piece was extracted, the uterine perforation primarily repaired and an end to end anastomosis was done after resection of about 15 cm of the non-viable portion of the bowel. Thorough peritoneal lavage and mopping was done and the abdomen closed leaving behind two drains. Two units of whole blood were transfused in the immediate post-operative period and parenteral antibiotics were continued. Drains were removed on post-operative day 4. Recovery was uneventful and patient was discharged on post-operative day 13. We also offered psychosocial support via counselling of both patient and family. Patient is doing fine after weekly follow up over the last 5 weeks.

## Discussion

Global reports suggest a declining trend in the incidence of abortion, though the proportion of unsafe abortions is gradually increasing in low and middle income countries (LMIC) [2]. According to the World Health Organization (WHO), every 8 min a woman in a LMIC dies due to complications from unsafe abortions, thus accounting for a leading cause of maternal mortality. In LMICs, 55 % of abortions are unsafe in contrast to 3 % in high income countries [1, 2, 9]. According to the WHO, unsafe abortion remains a public health issue despite being one of the easiest preventable causes of maternal mortality and morbidity [3].

Uterine perforation and bowel injuries are the major complications following unsafe abortion. The incidence of uterine perforation reported elsewhere varies from 0.4 to 15 per 1000 abortions [3, 4]. Although most uterine perforations at the time of curettage during first trimester abortion go unrecognized and untreated [10], serious complications like hemorrhage, septicemia, septic shock, and visceral injuries do occur. This was the case with our patient. Moreover, in our setting, the majority of such events are concealed initially, thereby favouring aggravation of existing complications.

These complications are said to occur partly as a result of the low resource setting, use of unsterile and inappropriate equipment for termination of pregnancy. In rural settings in LMICs, the situation is worrisome as most of these untrained practitioners insert foreign bodies into the uterus to disrupt pregnancy which can damage the uterus and internal organs including bowel [8, 10, 11] as was observed in our patient. In most instances, these are consequences of unwanted pregnancies being terminated by inexperienced individuals, usually without the necessary aseptic measures in place, in a bid to keep the act hidden. This finally leads to greater risk of injuries, morbidity and mortality [10, 11].

Perforation of the uterus, bleeding, visceral injury, sepsis, and shock following an unsafe abortion can ultimately lead to death due to delay in presentation for adequate medical treatment. In our case, the uterus was perforated by unskilled personnel leading to intraperitoneal hemorrhage and septicemia. Amongst others, unsafe abortion has been associated with long-term complications such as vesico-vaginal and recto-vaginal fistulae, chronic pelvic inflammatory disease with consequent dyspareunia, dysmenorrhea and infertility [3, 12].

Despite the adverse outcome associated with unsafe abortions as in the present case, the low socioeconomic status of these rural women, being pregnant before marriage, fear of parents’ reaction, desire to complete education, religious factors, social stigma attached to abortion, and civil laws prohibiting abortion, partly account for these unsafe methods for termination of pregnancy by unskilled personnel with resultant threat to life [10].

Although sonography is a helpful adjunct in detecting retained products of conception, it is important to note that the diagnosis of uterine perforation and bowel injury is based primarily on clinical suspicion especially in resource poor settings where imaging modalities are not readily available. In this case, a detailed clinical assessment and a high index of suspicion were pivotal in making the diagnosis, to which surgical exploration provided the definitive diagnosis and treatment. The timely recognition and appropriate management of such complications can significantly reduce morbidity and mortality.

## Conclusion

Unsafe abortion is an important social and public health problem which accounts for a significant cause of maternal mortality and morbidity in LMICs. In these settings, the procedure is usually carried out by untrained persons. Proper health education, awareness about various methods of contraception and easy accessibility to safer methods of abortion should be promoted. A high index of suspicion and detailed clinical assessment are paramount especially in rural settings with limited imaging modalities in order to prevent the ensuing and deleterious complications.

## Consent

Written informed consent was obtained from the patient for publication of this case report and accompanying images.
